# First evidence of denitrification vis-à-vis monsoon in the Arabian Sea since Late Miocene

**DOI:** 10.1038/srep43056

**Published:** 2017-02-21

**Authors:** Shubham Tripathi, Manish Tiwari, Jongmin Lee, Boo-Keun Khim, IODP Expedition 355 Scientists, Dhananjai K. Pandey, Peter D. Clift, Denise K. Kulhanek, Sergio Andò, James A.P. Bendle, Sophia Aharonovich, Elizabeth M. Griffith, Gundiga P. Gurumurthy, Annette Hahn, Masao Iwai, Anil Kumar, A. Ganesh Kumar, Hannah M. Liddy, Huayu Lu, Mitchell W. Lyle, Ravi Mishra, Tallavajhala Radhakrishna, Claire M. Routledge, Rajeev Saraswat, Rakesh Saxena, Giancarlo Scardia, Girish K. Sharma, Arun D. Singh, Stephan Steinke, Kenta Suzuki, Lisa Tauxe, Zhaokai Xu, Zhaojie Yu

**Affiliations:** 1 National Centre for Antarctic and Ocean Research, Vasco-da-Gama, 403804, Goa, India; 2 Department of Oceanography, Pusan National University, Busan, 46241, Korea; 3 Department of Geology and Geophysics, Louisiana State University, E253 Howe-Russell-Kniffen, Geoscience Complex, Baton Rouge, LA 70803, USA; 4 International Ocean Discovery Program, Texas A&M University, 1000 Discovery Drive College Station TX 77845, USA; 5 Department of Earth and Environmental Sciences, University of MilanoBicocca, Piazza della Scienza 4, 20126 Milan, Italy; 6 School of Geography, Earth and Environmental Sciences, University of Birmingham, Edgbaston, Birmingham B15 2TT United Kingdom; 7 Department of Earth and Planetary Sciences, Faculty of Science and Engineering, Macquarie University Level 2, The Australian Hearing Hub, 16 University Avenue, Sydney NSW 2109, Australia; 8 Department of Earth and Environmental Sciences, University of Texas at Arlington, Geosciences Building, Room 107, 500 Yates Street, Arlington TX 76019 USA; 9 Manipal Centre for Natural Sciences, Manipal University, Dr. T.M.A. Pai Planetarium Building, Manipal 576104, India; 10 MARUM, University of Bremen, Leobener Strasse 28359 Bremen, Germany; 11 Center for Advanced Marine Core Research/Natural Science Cluster, Kochi University, 2-5-1 Akebono-cho, Kochi 780-8520, Japan; 12 Wadia Institute of Himalayan Geology, 33 GMS Road, Dehradun Uttrakhand 248001, India; 13 Marine Biotechnology Department, National Institute of Ocean Technology, Velachery-Tambaram Main Road Pallikaranai, Chennai 600100, India; 14 Department of Earth Sciences, University of Southern California, 3651 Trousdale Parkway Los Angeles CA 90089, USA; 15 School of Geographical and Oceanographical Sciences, Nanjing University, 163 Xianlin Avenue, Nanjing 210023, China; 16 College of Earth, Ocean and Atmospheric Sciences, Oregon State University, 104 CEOAS Administration Building, Corvallis OR 97331 USA; 17 Geosciences Division, National Centre for Earth Science Studies, Aakkulam, Trivandrum 695031, India; 18 University College London, Gower Street, London WC1E 6BT, United Kingdom; 19 Geological Oceanography Division, National Institute of Oceanography, Dona Paula, Goa 403004, India; 20 ONGC 11 High, Bandra-Sion Link Road, Mumbai 400017, India; 21 Instituto de Geociências e Ciências Exatas, Universidade Estadual Paulista, 1515 Avenida 24-A Rio Claro SP 13506-900, Brazil; 22 Department of Geology, Kumaun University, Nainital 263002, India; 23 Department of Geology, Banaras Hindu University, Varanasi, Uttar Pradesh 221005, India; 24 Department of Geological Oceanography, College of Ocean and Earth Sciences, Xiamen University, Xiping Building, Xiang’an South Road, Xiang’an District, Xiamen 361102, China; 25 Graduate School of Environmental Science, Hokkaido University, N10W5, Kita-ku Sapporo 060-0810, Japan; 26 Scripps Institution of Oceanography, 9500 Gilman Drive, La Jolla CA 92093-0220, USA; 27 Key Laboratory of Marine Geology and Environment, Institute of Oceanology, Chinese Academy of Sciences 7 Nanhai Road, Qingdao Shandong 266071, China; 28 Laboratoire Géosciences Paris-Sud (GEOPS, UMR8148-CNRS) Université de Paris-Sud (Orsay) Bâtiment 504 91405, Orsay Cedex, France

## Abstract

In the Arabian Sea, South Asian monsoon (SAM)-induced high surface water productivity coupled with poor ventilation of intermediate water results in strong denitrification within the oxygen minimum zone (OMZ). Despite the significance of denitrification in the Arabian Sea, we have no long-term record of its evolution spanning the past several million years. Here, we present the *first* record of denitrification evolution since Late Miocene (~10.2 Ma) in the Eastern Arabian Sea, where the SAM generates moderate surface water productivity, based on the samples retrieved during the International Ocean Discovery Program (IODP) Expedition 355. We find that (i) the SAM was persistently weaker from ~10.2 to 3.1 Ma; it did not intensify at ~8 Ma in contrast to a few previous studies, (ii) on tectonic timescale, both the SAM and the East Asian Monsoon (EAM) varied synchronously, (iii) the first evidence of denitrification and productivity/SAM intensification was at ~3.2–2.8 Ma that coincided with Mid-Pliocene Warm Period (MPWP), and (iv) the modern strength of the OMZ where denitrification is a permanent feature was attained at ~1.0 Ma.

Oxygen minimum zones (OMZs) - the regions of dissolved oxygen deficient (O_2_ < 20 μM) water located in the tropical oceans - have been proposed to expand in the present scenario of global warming^1^,^2^. OMZs play a significant role in producing N_2_O - a powerful greenhouse gas through the process of denitrification (a process by which nitrate and nitrite are reduced to nitrogen gas) when the dissolved O_2_ levels fall below 1 μΜ^3^. A perennial OMZ develops between 150 and 1000 m water depth in the Arabian Sea due to various natural factors such as high surface water productivity and reduced ventilation of intermediate water^4^. The anoxic zones of these OMZs occupy only ~0.8% of the world ocean but are responsible for the highest production of N_2_ through denitrification (~35% of the global production) out of which the Arabian Sea contributes the largest proportion (~17% of global N_2_ production)^5^. The balance between nitrogen fixation and its removal through N_2_ production is a key to carbon assimilation by primary production and CO_2_ regulation in the atmosphere^3^,^6^. In the Arabian Sea, most of the studies have examined denitrification variability over the past 100 kyr or younger; the longest record available goes back to 1 Ma in the Western Arabian Sea^7^. Hence, there is a lack of information regarding the long-term evolution of denitrification spanning the past several million years, especially from the Eastern Arabian Sea. Here, we examine samples from Site U1456 in the Eastern Arabian Sea retrieved during the IODP Expedition 355^8^ (Fig. 1).

To reveal the long-term OMZ variability and its coupling with surface water productivity, we analyzed multiple isotopic and geochemical proxies viz. nitrogen and carbon isotopic ratios (δ^15^N and δ^13^C), total organic carbon and total nitrogen (TOC and TN) concentrations, and carbon to nitrogen (C/N) weight ratio of sedimentary organic matter (SOM).

## Study Area

**Figure 1:**
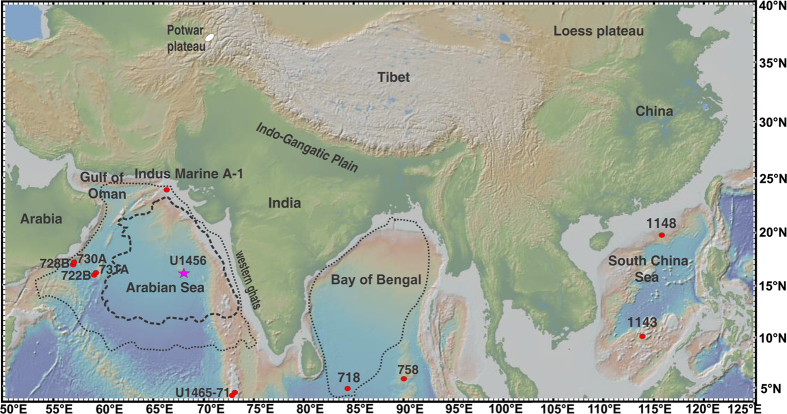
Locations of the IODP Expedition 355 Site U1456 in the Eastern Arabian Sea (3640 m of water depth, 16°37.28′N, 68°50.33′E) denoted by pink star^8^. The red circles represent ODP and IODP sites in the Arabian Sea^7^,^27^,^28^,^29^,^32^, Bay of Bengal^33^,^34^ and South China Sea^38^,^41^, which have been discussed in the present study. The white patch represents Potwar plateau^31^. The thin dotted curves in the Arabian Sea and the Bay of Bengal show modern anoxia^1^ based on WOA2005 climatology. The thick black dotted curve in the Arabian Sea represents the approximate extent of denitrification zone^42^ (Figure created using GeoMapApp3.6.0, www.geomapapp.org).

Site U1456 is located at 16°37.28′N, 68°50.33′E in the Eastern Arabian Sea (EAS) (Fig. 1), ~475 km away from the Indian coast, and ~820 km from the modern mouth of the Indus River, and within the Laxmi Basin which is flanked by the Laxmi Ridge to the west and the Indian continental shelf to the east. The Laxmi Basin is characterized by a 200–250 km wide depression that runs in a northwest–southeast direction parallel to the west coast of India^8^. The site is situated at a water depth of 3640 m, which lies well above the modern lysocline (~3800 m) in the Arabian Sea^8^. Three distinct water masses identified by Rochford^9^ in the Arabian Sea are Arabian Sea High Salinity Water (~50 m to 75 m) (ASHSW), Persian Gulf Water (~25 m to 70 m) (PGW), and Red Sea Water (~600 m to 900 m) (RSW)^10^. ASHSW shows greater seasonal variability than PGW and RSW and is considered as the main source of oxygen in the Western Arabian Sea (WAS)^9^,^11^. Thus, the subsurface denitrification intensity in the WAS is controlled by the surface productivity as well as the supply of oxygen from the water masses^11^. However, in the EAS, the subsurface denitrification is expected to be controlled mainly by the extent of surface productivity^12^. An Argo float-based study in the Arabian Sea revealed the presence of high salinity water with inter-seasonal to inter-annual variability^13^. The vertical mixing of PGW and RSW between ~250 m to ~800 m result in the formation of the Arabian Sea Intermediate Water^14^. The deep water masses of the Indian Ocean comprise Antarctic Bottom Water (AABW), Circumpolar Deep Water (CDW), and Indian Deep Water (IDW). IDW forms in the Indian Ocean itself by the process of diffusion and upwelling and is characterised by low oxygen content and relatively enriched nutrients because of its aging^15^. The present-day bottom water in the Arabian Sea flows northward and upwells into the layer of North Indian Deep Water (~1500–3500 m)^16^.

## Results and Discussion

**Figure 2:**
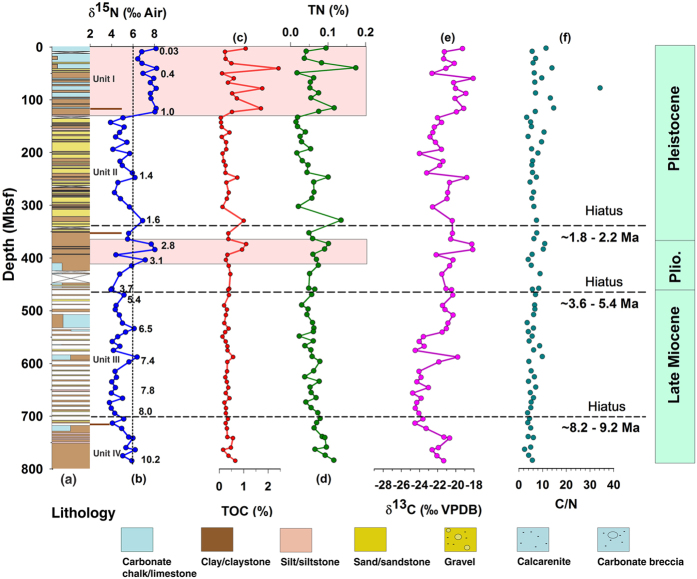
Record of denitrification, surface water productivity, and provenance of the Sedimentary Organic Matter (SOM) in the Eastern Arabian Sea since Late Miocene. (**a**) Lithostratigraphy of site U1456, (**b**) denitrification variability (δ^15^N of SOM), (**c,d**) paleoproductivity variability (weight percent total organic carbon [TOC] and total nitrogen [TN] of SOM), (**e,f**) SOM provenance indicators (δ^13^C and C/N ratio). The coloured, rectangular boxes show the intensified OMZ coupled with surface water productivity when denitrification occurred in the basin. The horizontal dotted lines indicate the position of the hiatuses. The vertical dashed line over panel ‘**b**’ show denitrification threshold and horizontal brown lines separates different lithological units. The age data (in Ma) at Site U1456, shown by the Indo-Arabic numerals in ‘panel **b**’, are based on calcareous nannofossil and planktonic foraminifera biostratigraphy, together with magnetostratigraphy^8^.

The drilled section at Site U1456 is divided into four lithologic units based on a variety of sediment properties (Fig. 2a); Unit I (~121 m thick and Pleistocene nannofossil ooze interbedded with very thin turbidites), Unit II (~240 m thick and late Pliocene to early Pleistocene sand and silt), Unit III (~370 m thick and late Miocene to late Pliocene clay/claystone, sand/sandstone, nannofossil chalk, and nannofossil-rich claystone), and Unit IV (~380 m thick and older than late Miocene claystone, calcarenite, calcilutite, and conglomerate/breccia). These lithologies are characterized by different mineralogical and geochemical properties^8^.

Since the drilled core is very long (1109.4 m) and the site is quite deep (3640 m)^8^, the isotopic ratios of the SOM should be evaluated for the diagenetic alterations related to the lithology. Diagenesis of the organic matter begins within the photic zone of the water column, which continues during sinking. It further maintains unceasingly within the bioturbated mixed layer of sediment (a few cm to ~10 cm depth) and only a few percent (1 to 0.01%) of organic matter is finally buried/preserved in the sediment^17^. Although microbial activity has been found even up to several hundred meters deep into the sedimentary sequence^18^, diagenesis reduces significantly with increasing depth. Popp *et al*.^19^ suggested that despite the loss of organic matter due to remineralization, the δ^13^C of SOM remains almost unchanged with increasing depth. Similarly, a very small δ^15^N offset was found between core top sediments and sinking particles in the equatorial Pacific region; the loss of organic matter due to diagenesis in the upper section of the core top shows no corresponding δ^15^N change^11^. Core top studies from the Western Arabian Sea reported no correlation between TN and δ^15^N, which indicates that diagenesis does not affect δ^15^N variation^7^. We also obtain no relationship between TN and δ^15^N (r^2^ = 0.19; Supplementary Fig. 1). Thus, diagenesis appears to cause no significant alteration in δ^13^C and δ^15^N values of SOM at Site U1456.

The C/N ratio of marine organic matter generally ranges from 8 to 10^20^. Terrestrial organic matter predominantly consists of compounds like cellulose and lignin with much low nitrogen content. The C/N ratios of land-derived organic matter, therefore, are much high in the range between 20 and 100^20^. The mean δ^13^C values of the marine organic matter, C4, and C3 plants are about −21‰, −13‰, and −27‰, respectively^21^. The C/N ratio together with δ^13^C of SOM has been widely used to determine the origin of organic matter^20^. At Site U1456, the δ^13^C values vary from −18‰ to −25‰ and most of the C/N ratios range from 6 to 10, indicating that SOM is mostly of marine origin (Fig. 2e,f and Supplementary Fig. 2).

Based on surface sediment analysis of more than 100 locations in the Central and Eastern Arabian Sea (most of them are located in the Eastern Arabian Sea), the δ^15^N values of SOM have been found to vary from 6‰ to 11‰^22^. In most of the oxygenated basins, the δ^15^N values do not exceed 6‰ while those from the oxygen deficient basins are highly enriched with mostly higher than 6‰^7^,^22^,^23^,^24^. Thus, the periods with δ^15^N values higher than 6‰ may signify denitrification associated with strong OMZ. At Site U1456, the δ^15^N values of SOM vary between 2.4‰ to 8.2‰ (Fig. 2b). The maximum TOC and TN values are 2.42% and 0.17%, respectively (Fig. 2c,d). The Mid-to Late Pliocene (~3.2 to 2.7 Ma) is characterized by high δ^15^N values (>6‰) along with high TOC and TN values, indicating denitrification/strong OMZ (Fig. 2). Another period of denitrification/OMZ intensification (δ^15^N > 6‰) takes place from ~1.0 Ma to the core top (0.03 Ma) (Fig. 2b). During these periods of intense denitrification, the surface water productivity indicators viz. TOC and TN contents also represent an increasing trend (Fig. 2c,d). Intense wind-induced productivity and particle flux occur in the Arabian Sea during the monsoon seasons^25^. Modern climatological chlorophyll *a* data show that the surface water productivity in the Eastern Arabian Sea is driven by both the summer and the winter monsoons^26^. Thus, surface water productivity variability in the Eastern Arabian Sea is a manifestation of the SAM variability, which can be linked to denitrification/OMZ intensification.

**Figure 3:**
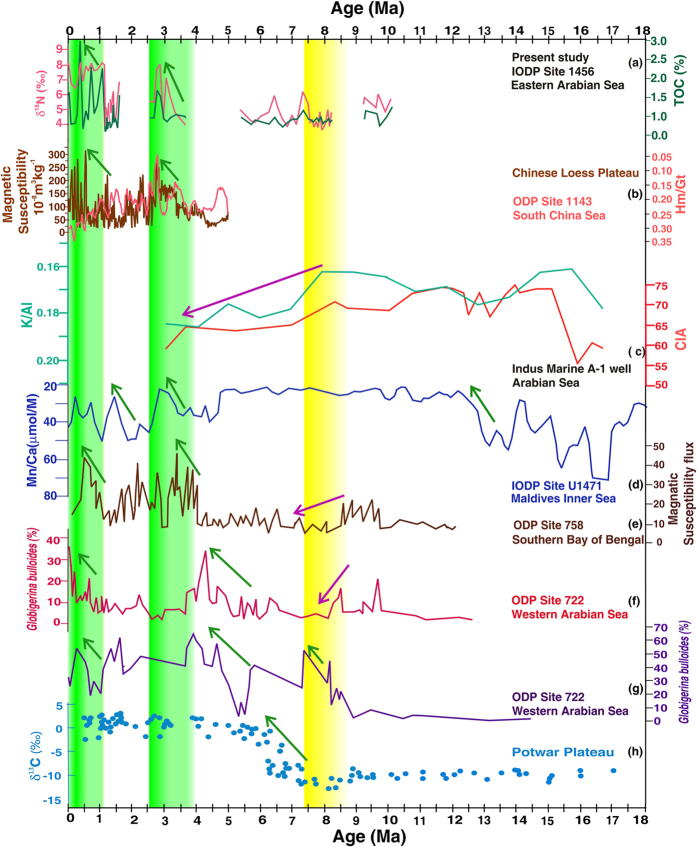
Comparative records of the South Asian Monsoon and East Asian Monsoon since Mid-Miocene. (**a**) δ^15^N and total organic carbon (TOC) from IODP site U1456, (**b**) Magnetic susceptibility record^37^ of Chinese loess plateau and Hm/Gt (40 point moving average) from the South China Sea ODP site 1143^38^, (**c**) Chemical Index of Weathering (CIA) from the Indus river fan^32^, (**d**) Mn/Ca record from the Maldives inner Sea^30^ (**e**) Magnetic susceptibility record of the southern Bay of Bengal ODP site 758^34^, (**f**) *G. bulloides* abundance from ODP site 722^29^, (**g**) *G. bulloides* abundance from ODP site 722^27^, and (**h**) δ^13^C of calcretes from the Potwar Plateau^31^. The green arrows represent the strengthening of monsoon and the purple indicate the weakening of monsoon. The yellow band marks the arid period when many of the studies including the present study show the weakened monsoon while the green bands indicate the periods of strengthened monsoon.

The origin and evolution of the SAM are still a topic of debate. According to the previous hypothesis based on a study from the Western Arabian Sea (Ocean Drilling Program (ODP) Site 722), the initiation/intensification of the SAM occurred at around 8.5 Ma and continued until 6 Ma^27^ (Fig. 3g). Another study from the same ODP Site 722 shows that the onset of the SAM took place at ~12.9 Ma and a major intensification occurred at ~7 Ma^28^. In contrast, a decrease in *G. bulloides* abundance was found at 8.5 Ma (Fig. 3f) from the ODP Site 722 implying reducing SAM^29^. A recent study from the inner seas of the Maldives (IODP sites U1465-71) postulates a proto-monsoon from 25–12.9 Ma and an abrupt increase in the monsoon at ~12.9 Ma^30^ (Fig. 3d). The δ^13^C values of paleosols from the Siwalik Group sediments in the northern Pakistan spanning the past 18 Myr showed a marked shift from C-3 to C-4 dominated plants at ~7.4 Ma, which may be associated with SAM inception and again the flood plains were mostly occupied by C-4 grassland in Plio-Pleistocene^31^ indicating monsoon intensification (Fig. 3h). Recent records of Himalayan weathering represented by the chemical index of alteration (CIA) and K/Al ratios (Fig. 3c) demonstrated that SAM attained the maximum strength at 15 Ma, remained high until 10.5 Ma, gradually weakened until ~3.5 Ma, and again increased from the Late Pliocene to Pleistocene^32^. The Sr isotope and clay mineral data also suggested weaker SAM after 8 Ma^33^. Our record from Site U1456 spans ~10.2 to 0.03 Ma, but includes several hiatuses dated to ~8.2–9.2 Ma, ~3.7–5.4 Ma, and ~1.6–2.2 Ma^8^. Nevertheless, we interpret that surface water productivity in the Eastern Arabian Sea was low from 10.15 Ma to 3.2 Ma as evident from uniformly low values of TOC and TN (3a and 2b). Additionally, during this period, the δ^15^N did not reach the threshold value (~6‰) indicative of denitrification (Fig. 3a). This implies that neither the surface water productivity (TOC, TN) nor the OMZ intensity supports any major intensification in SAM strength from ~10 to ~3.2 Ma, which is also documented in the different regions (the South China Sea, the Northern Arabian Sea and the Bay of Bengal)^32^,^34^. These studies^32^,^34^ reported that SAM and EAM were reduced more or less in parallel albeit with a time-lag; the EAM started declining at ~10 Ma while the SAM began decreasing at ~8 Ma. But, we find that the SAM was weak at ~10 Ma indicating that EAM and SAM varied in consonance, without any apparent time lag, on tectonic timescale. This Late Miocene reduction in monsoon strength could be a result of global cooling after the Middle Miocene Climatic Optimum^35^. At around 8 Ma, δ^15^N values vary between 3.7‰ to 5.8‰, i.e., the OMZ was not intense enough to cause denitrification and the surface water productivity was diminished (Figs 2 and 3a), which implies that SAM did not intensify at ~8 Ma.

During the study period, for the first time, the OMZ intensified to the level that denitrification takes place was at ~3.2–2.8 Ma (Fig. 2b). During this period, the surface water productivity (Fig. 2c,d) was also enhanced, indicating stronger SAM, which coincides with MPWP^36^. Earlier studies, based on magnetic susceptibility (Chinese Loess Plateau, Fig. 3b; southern Bay of Bengal, Fig. 3e) and hematite to goethite ratio (Hm/Gt, South China Sea, Fig. 3b), also reported the enhanced SAM and EAM during ~3.6–2.6 Ma^34^,^37^,^38^. A new magnetostratigraphy study from Chinese Loess Plateau spanning from ~8.2 Ma to 2.6 Ma documented long-term East Asian Summer Monsoon (EASM) intensification. Both proxy, as well as numerical climate model assessment, show that the Antarctic glaciation was an important driver for the long-term trend of late Miocene-Pliocene EASM intensification^39^. To examine the responsible mechanisms, a modeling experiment, using the NCAR climate model CCM3, with idealized Himalayan-Tibetan Plateau elevations explains the observed increase of the EAM as a result that the Himalayan-Tibetan Plateau attained modern extension along its eastern and northern margins^34^. It was speculated that it might not have affected the SAM circulation pattern^34^. The present study, based on the multi-proxy records, suggests that the SAM was also enhanced in parallel with the EAM and therefore the intensification can be ascribed to the same mechanism. A recent review^40^ investigated the role of the Tibet Plateau in affecting SAM, and found that it simply acts as a physical barrier for northerly cool, dry winds. Its role as an elevated heat source is of secondary importance in affecting the SAM. EAM dynamics is also affected by the Tibet Plateau, which is located in the path of subtropical jet streams^40^. The increase in both the EAM and SAM during ~3.6–2.6 Ma could have resulted in the increased weathering and organic carbon burial, as evident by higher TOC (Fig. 2c), leading to atmospheric CO_2_ drawdown that would have possibly contributed to Northern Hemisphere Glaciation (NHG) at 2.7 Ma^40^. Thereafter, from 2.8 Ma to ~1.0 Ma, δ^15^N values as well as the surface water productivity declined in parallel, indicating relatively weaker SAM. Previous studies also reported the weakened EAM and SAM after ~2.6 Ma^34^,^36^,^37^, confirming our results, which coincides with the onset of NHG. Finally, the OMZ reached its modern strength, i.e., denitrification became a permanent feature, at about ~1.0 Ma closely following the enhanced surface water productivity. It implies that SAM intensified from ~1.0 Ma as reported in earlier studies viz. the enhanced sedimentation rate in the Indus Fan^32^, the increased chemical weathering from the Bengal Fan^33^ and the South China Sea^41^, the rise of magnetic susceptibility (Fig. 3b) and mean sediment flux from the Indian Ocean^38^.

## Methods

The samples used in the present study were obtained onboard the *JOIDES Resolution.* 5–15 cm long whole-round core sections at the interval of every core or every alternate core were squeezed using titanium steel squeezing device to obtain the interstitial water. The remaining sediments are named ‘squeeze cake’. The samples were dried to remove the moisture at 45 °C before processing. Around 10 to 20 g of sediment aliquots were taken for further analysis. Dried samples were finely grounded for homogenization. Homogeneous samples were divided into two batches for geochemical and isotopic analyses - (i) 2 N HCl treatment for total organic carbon (TOC) and *δ*^13^C measurement and (ii) untreated for determination of total nitrogen (TN) content and *δ*^15^N values. 20 ml of 2 N HCl solution was added to 5–10 g of fine sediment powder. The mixture was shaken mechanically and allowed to stand for ~12 hours. The sample was then washed with ultrapure demineralized water and approximately 25 mg of treated sample was used for TOC and *δ*^13^C analysis. For TN and *δ*^15^N measurement, approximately 40 mg of bulk ground sediment was used. The δ^15^N and δ^13^C values were determined using isotope ratio mass spectrometer coupled with an element analyzer at Marine Stable Isotope Lab, National Centre for Antarctic and Ocean Research, Goa, India and Department of Oceanography, Pusan National University, Busan, Korea. The standard used was ammonium sulfate (IAEA-N-1) and cellulose (IAEA-CH-3). The analytical precision for δ^15^N and δ^13^C is ±0.12‰ and ±0.06‰, respectively. Similarly, TN and TOC were determined using sulfanilamide as the standard. The analytical precision for TN and TOC is ±0.63% and ±0.84%, respectively.

### Data Availability

The data used in this study are included in the supplementary information files.

## Additional Information

**How to cite this article:** Tripathi, S. *et al*. First evidence of denitrification vis-à-vis monsoon in the Arabian Sea since Late Miocene. *Sci. Rep.* **7**, 43056; doi: 10.1038/srep43056 (2017).

**Publisher's note:** Springer Nature remains neutral with regard to jurisdictional claims in published maps and institutional affiliations.

## Supplementary Material

Supplementary Figures

Supplementary Dataset
