# Supplementary material for: First evidence of denitrification vis-à-vis monsoon in the Arabian Sea since Late Miocene
Source: Sci Rep. 2017 Feb 21;7:43056. doi: 10.1038/srep43056 (PMC5318868; doi:10.1038/srep43056)
Supplement: Supplementary Figures [file srep43056-s1.pdf]

SUPPLEMENTARY MATERIAL

First evidence of denitrification vis-à-vis monsoon in the Arabian Sea since Late Miocene

Shubham Tripathi[1], Manish Tiwari*[1], Jongmin Lee[2], Boo-Keun Khim[#2], and IODP Expedition 355 Scientists

[1]National Centre for Antarctic and Ocean Research, Vasco-da-Gama, 403804, Goa, India

[2]Department of Oceanography, Pusan National University, Busan, 46241, Korea

* *Corresponding Author*: Manish Tiwari; email: manish@ncaor.gov.in; Phone: +91-832-2525638; Fax: +91-832-2520877

\# *Co-corresponding Author*: Boo-Keun Khim; email: bkkhim@pusan.ac.kr; Phone: +82-51-5102212; Fax: +82-51-5812963

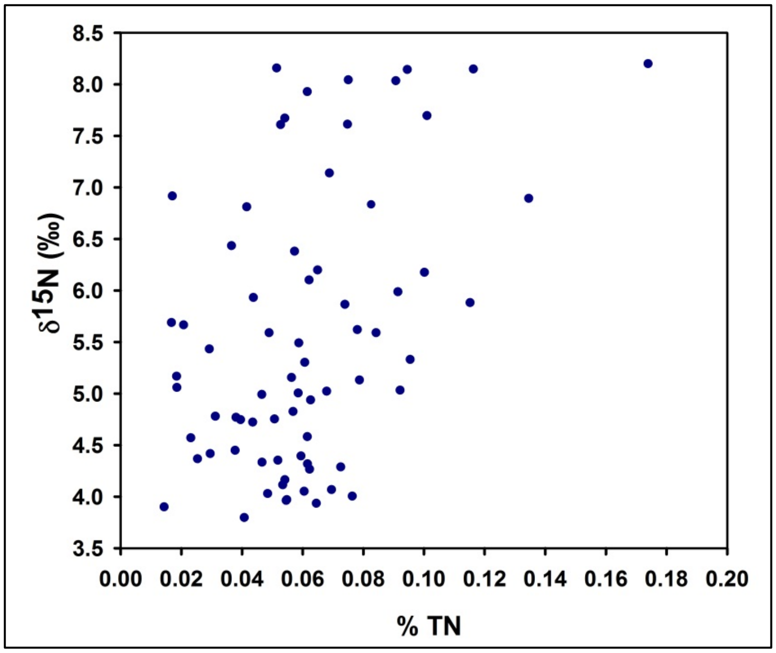

Supplementary Figure 1: $\delta^{15}N$ of sedimentary organic matter (SOM) versus weight percent total nitrogen of SOM of the squeeze cake samples at Site U1456 used in the present study. There is no relationship between $\delta^{15}N$ and %TN ($r^2 = 0.19$) indicating that diagenesis did not alter the $\delta^{15}N$ values.

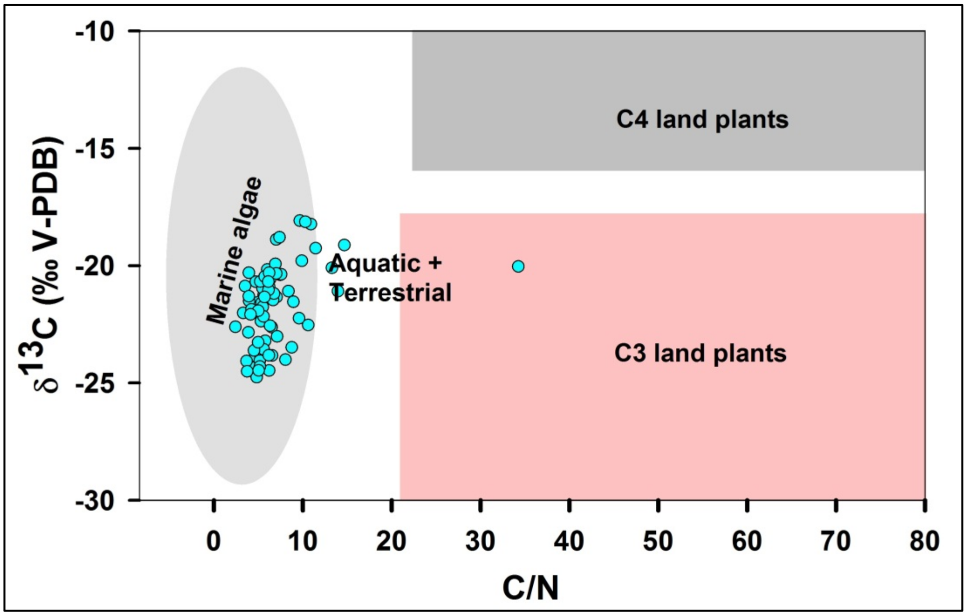

Supplementary Figure 2: The $\delta^{13}C$ versus C/N ratios as indicator of the provenance of the organic matter. Shaded areas approximately show the range of the $\delta^{13}C$ values for that particular group.
