# Supplementary material for: First evidence of denitrification vis-à-vis monsoon in the Arabian Sea since Late Miocene
Source: Sci Rep. 2017 Feb 21;7:43056. doi: 10.1038/srep43056 (PMC5318868; doi:10.1038/srep43056)
Supplement: Supplementary Dataset [file srep43056-s2.doc]

**SUPPLEMENTARY DATA**

**First evidence of denitrification vis-à-vis monsoon in the Arabian Sea since Late Miocene**

Shubham Tripathi^1^, Manish Tiwari*^1^, Jongmin Lee^2^, Boo-Keun Khim^#2^, and IODP Expedition 355 Scientists

^1^National Centre for Antarctic and Ocean Research, Vasco-da-Gama, 403804, Goa, India

^2^Department of Oceanography, Pusan National University, Busan, 46241, Korea

* *Corresponding Author*: Manish Tiwari; email: manish@ncaor.gov.in; Phone: +91-832-2525638; Fax: +91-832-2520877

# *Co-corresponding Author*: Boo-Keun Khim; email: bkkhim@pusan.ac.kr; Phone: +82-51-5102212; Fax: +82-51-5812963

**Table.1 Isotope and Geochemical data used in the present study.**

| **Depth (mbsf)** | **Age (Ma)** | **δ^15^N (‰)** | **TN (%)** | **TOC (%)** | **δ^13^C (‰)** | **C/N** |
| --- | --- | --- | --- | --- | --- | --- |
| 2.95 | 0.03 | 8.14 | 0.09 | 1.08 | -19.26 | 11.44 |
| 8.95 | 0.08 | 6.81 | 0.04 | 0.23 | -21.26 | 5.60 |
| 22.45 | 0.21 | 6.44 | 0.04 | 0.26 | -21.34 | 7.05 |
| 30.95 | 0.28 | 6.84 | 0.08 | 0.50 | -20.17 | 6.01 |
| 40.45 | 0.37 | 8.20 | 0.17 | 2.43 | -21.08 | 13.96 |
| 49.95 | 0.46 | 6.92 | 0.02 | 0.11 | -22.62 | 6.49 |
| 59.45 | 0.55 | 7.93 | 0.06 | 0.59 | -18.08 | 9.67 |
| 67.45 | 0.62 | 7.61 | 0.05 | 0.35 | -20.25 | 6.71 |
| 78.45 | 0.72 | 8.16 | 0.05 | 1.76 | -20.03 | 34.26 |
| 87.95 | 0.81 | 7.61 | 0.07 | 0.52 | -18.89 | 7.01 |
| 97.45 | 0.9 | 7.67 | 0.05 | 0.72 | -20.09 | 13.28 |
| 116.42 | 1.07 | 8.15 | 0.12 | 1.71 | -19.12 | 14.68 |
| 122.95 | 1.12 | 8.04 | 0.08 | 0.52 | -19.93 | 6.92 |
| 134.12 | 1.15 | 5.06 | 0.02 | 0.06 | -22.02 | 3.29 |
| 142.97 | 1.17 | 3.90 | 0.01 | 0.07 | -21.56 | 4.96 |
| 151.65 | 1.19 | 5.17 | 0.02 | 0.10 | -22.37 | 5.30 |
| 162.05 | 1.21 | 4.75 | 0.04 | 0.42 | -22.54 | 10.61 |
| 170.45 | 1.23 | 4.37 | 0.03 | 0.10 | -22.84 | 3.88 |
| 180.56 | 1.26 | 5.43 | 0.03 | 0.28 | -22.24 | 9.58 |
| 193.95 | 1.29 | 4.12 | 0.05 | 0.29 | -21.58 | 5.37 |
| 201.85 | 1.31 | 5.69 | 0.02 | 0.13 | -24.01 | 8.05 |
| 216.65 | 1.34 | 4.78 | 0.03 | 0.18 | -21.41 | 5.80 |
| 224.55 | 1.36 | 4.99 | 0.05 | 0.26 | -21.76 | 5.49 |
| 238.65 | 1.39 | 5.93 | 0.04 | 0.25 | -23.21 | 5.76 |
| 247.55 | 1.41 | 6.18 | 0.10 | 0.74 | -18.79 | 7.38 |
| 256.91 | 1.43 | 4.58 | 0.06 | 0.29 | -20.69 | 4.71 |
| 275.96 | 1.48 | 4.27 | 0.06 | 0.39 | -20.89 | 6.21 |
| 287.55 | 1.51 | 4.83 | 0.06 | 0.31 | -20.96 | 5.47 |
| 303.15 | 1.54 | 5.67 | 0.02 | 0.13 | -22.57 | 6.32 |
| 329.29 | 1.6 | 6.89 | 0.13 | 1.00 | -20.39 | 7.44 |
| 353.3 | 2.57 | 5.59 | 0.05 | 0.37 | -20.37 | 7.56 |
| 364.2 | 2.69 | 5.49 | 0.06 | 0.37 | -20.56 | 6.33 |
| 373.6 | 2.78 | 7.70 | 0.10 | 1.10 | -18.23 | 10.91 |
| 384 | 2.89 | 8.04 | 0.09 | 0.93 | -18.13 | 10.29 |
| 393.9 | 2.99 | 4.40 | 0.06 | 0.33 | -22.17 | 5.58 |
| 403.7 | 3.09 | 7.14 | 0.07 | 0.27 | -20.30 | 3.94 |
| 414.8 | 3.21 | 5.87 | 0.07 | 0.39 | -20.68 | 5.22 |
| 430.6 | 3.37 | 4.75 | 0.05 | 0.45 | -21.54 | 8.94 |
| 458.41 | 3.65 | 4.03 | 0.05 | 0.41 | -21.09 | 8.38 |
| 460.2 | 3.67 | 3.94 | 0.06 | 0.37 | -20.46 | 5.70 |
| 470.21 | 5.4 | 5.16 | 0.06 | 0.39 | -20.33 | 7.01 |
| 490.04 | 5.73 | 4.42 | 0.03 | 0.20 | -21.47 | 6.65 |
| 497.81 | 5.86 | 4.33 | 0.05 | 0.31 | -21.19 | 6.75 |
| 508.14 | 6.04 | 4.72 | 0.04 | 0.27 | -20.30 | 6.21 |
| 523.49 | 6.29 | 5.01 | 0.06 | 0.20 | -20.87 | 3.50 |
| 533.82 | 6.47 | 6.10 | 0.06 | 0.38 | -21.04 | 6.15 |
| 540.55 | 6.58 | 5.30 | 0.06 | 0.24 | -21.50 | 3.97 |
| 549.14 | 6.72 | 4.57 | 0.02 | 0.13 | -23.57 | 5.61 |
| 558.4 | 6.88 | 4.05 | 0.06 | 0.26 | -24.04 | 4.32 |
| 567.39 | 7.03 | 4.77 | 0.04 | 0.33 | -23.49 | 8.76 |
| 575.3 | 7.16 | 4.16 | 0.05 | 0.34 | -24.47 | 6.21 |
| 587.82 | 7.37 | 6.38 | 0.06 | 0.57 | -19.79 | 9.88 |
| 596.99 | 7.53 | 5.62 | 0.08 | 0.33 | -21.87 | 4.22 |
| 615.02 | 7.6 | 4.32 | 0.06 | 0.32 | -24.06 | 5.16 |
| 626.24 | 7.67 | 4.45 | 0.04 | 0.25 | -23.83 | 6.54 |
| 634.8 | 7.72 | 4.01 | 0.08 | 0.31 | -24.30 | 4.06 |
| 645.75 | 7.79 | 4.35 | 0.05 | 0.37 | -23.02 | 7.11 |
| 656.15 | 7.85 | 3.97 | 0.05 | 0.26 | -24.76 | 4.82 |
| 665.92 | 7.91 | 5.02 | 0.07 | 0.42 | -23.82 | 6.18 |
| 674.22 | 7.97 | 3.80 | 0.04 | 0.21 | -24.30 | 5.17 |
| 684.75 | 8.03 | 3.96 | 0.05 | 0.27 | -24.47 | 5.04 |
| 694.46 | 8.09 | 4.29 | 0.07 | 0.27 | -24.07 | 3.68 |
| 705.23 | 8.16 | 5.13 | 0.08 | 0.35 | -23.62 | 4.50 |
| 713.19 | 8.21 | 4.07 | 0.07 | 0.26 | -24.50 | 3.74 |
| 723.18 | 8.27 | 4.94 | 0.06 | 0.31 | -23.27 | 4.98 |
| 739.59 | 9.28 | 5.59 | 0.08 | 0.33 | -21.31 | 3.92 |
| 741.45 | 9.32 | 5.99 | 0.09 | 0.56 | -20.68 | 6.12 |
| 759.6 | 9.67 | 5.33 | 0.10 | 0.48 | -21.92 | 4.99 |
| 763.66 | 9.75 | 6.20 | 0.06 | 0.16 | -22.61 | 2.42 |
| 775 | 9.97 | 5.03 | 0.09 | 0.38 | -22.08 | 4.12 |
| 784.47 | 10.15 | 5.88 | 0.12 | 0.65 | -21.34 | 5.68 |

|  |  |  |  |  |  |
| --- | --- | --- | --- | --- | --- |
